# Decolonization of patients and health care workers to control nosocomial spread of methicillin-resistant *Staphylococcus aureus:* a simulation study

**DOI:** 10.1186/1471-2334-12-302

**Published:** 2012-11-14

**Authors:** Tatiana V Gurieva, Martin CJ Bootsma, Marc JM Bonten

**Affiliations:** 1 Julius Center for Health Sciences and Primary Care, University Medical Center Utrecht, Heidelberglaan 100, P.O.Box 85500, Utrecht, GA, 3508, The Netherlands; 2 Faculty of Science, Department of Mathematics, Utrecht University, Budapestlaan 6, P.O.box 80010, Utrecht, CD, 3584, The Netherlands; 3 Department of Medical Microbiology, University Medical Center Utrecht. Heidelberglaan 100, P.O.Box 85500, Utrecht, GA, 3508, The Netherlands

## Abstract

**Background:**

Control of methicillin-resistant *Staphylococcus aureus* (MRSA) transmission has been unsuccessful in many hospitals. Recommended control measures include isolation of colonized patients, rather than decolonization of carriage among patients and/or health care workers. Yet, the potential effects of such measures are poorly understood.

**Methods:**

We use a stochastic simulation model in which health care workers can transmit MRSA through short-lived hand contamination, or through persistent colonization. Hand hygiene interrupts the first mode, decolonization strategies the latter. We quantified the effectiveness of decolonization of patients and health care workers, relative to patient isolation in settings where MRSA carriage is endemic (rather than sporadic outbreaks in non-endemic settings caused by health care workers).

**Results:**

Patient decolonization is the most effective intervention and outperforms patient isolation, even with low decolonization efficacy and when decolonization is not achieved immediately. The potential role of persistently colonized health care workers in MRSA transmission depends on the proportion of persistently colonized health care workers and the likelihood per colonized health care worker to transmit. As stand-alone intervention, universal screening and decolonization of persistently colonized health care workers is generally the least effective intervention, especially in high endemicity settings. When added to patient isolation, such a strategy would have maximum benefits if few health care workers cause a large proportion of the acquisitions.

**Conclusions:**

In high-endemicity settings regular screening of health care workers followed by decolonization of MRSA-carriers is unlikely to reduce nosocomial spread of MRSA unless there are few persistently colonized health care workers who are responsible for a large fraction of the MRSA acquisitions by patients. In contrast, decolonization of patients can be very effective.

## Background

Methicillin resistant *Staphylococcus aureus* (MRSA) is an important cause of nosocomial infections. The dynamics of MRSA transmission in health-care settings is characterized by high fluctuations in patient prevalence within units, resulting from patient-to-patient spread and admissions of colonized patients. So far, almost all interventions have been based on implementing barrier precautions for patients with documented MRSA carriage [1,2], sometimes in combination with decolonization of carriage [3]. The evidence for the efficacy of patient isolation to control nosocomial spread of MRSA in high endemicity settings, though, is rather limited [4].

Especially for MRSA, health care workers (HCWs) might be important in the nosocomial transmission dynamics. First, temporary contaminated hands of HCWs are important vectors for MRSA transmission [5], and appropriate hand hygiene is considered the key intervention to minimize this transmission mode [6]. Second, HCWs may become persistently colonized with MRSA [7], e.g., in the nose or on injured skin, and act as a constant source for MRSA transmission [7]. The obvious difference between both transmission roles is that hand hygiene will not clear persistent carriage.

MRSA eradication therapies using mupirocin and chlorhexidine were extremely efficacious in decolonizing HCWs [8]. If persistent carriage among HCW is an important source for MRSA transmission, decolonization of HCWs could be effective in controlling MRSA among patients. To the best of our knowledge, though, the relative contribution of persistently colonized HCWs in the epidemiology of MRSA endemicity has never been determined, and, consequently, there is no information on the possible effects of decolonizing persistently colonized HCWs. In contrast the efficacy of eradication therapies applied to patients during hospitalization seems to be low [8]. Clinical decision making for the most appropriate infection control strategy is frequently hampered by the absence of prospective comparisons of different control strategies. Moreover, even if performed, the importance of stochastic events in small populations, such as in hospitals, would necessitate long periods of follow-up. In the absence of empirical evidence, mathematical models may offer the best alternative to determine the optimal control strategy [9].

Here, we use a computer simulation model to quantify the effects of patient isolation and antimicrobial treatment of carriage for patients and HCWs, as part of an infection control program for MRSA with universal hospital admission screening. We aim to identify scenarios in which HCW decolonization could be considered a sensible intervention.

## Methods

### Patient and transmission dynamics

We use an extended version of a previously described stochastic simulation model [10]. The model contains three hospitals of 693 beds, each with an extramural population of 220,000 subjects. Patients are subdivided into “core group” and “non core group” patients, distinguished by hospitalization rates of once per year (core-group) and once per ten years (non-core group). On average 50% of the hospital population consists of “core group” patients.

Each hospital comprises two types of wards: five 9-bed Intensive Care Units (ICUs) and 36 18-bed regular wards. In ICUs the staff-patient ratio is 9:9, in regular wards 5:18. Besides HCWs confined to a single ward, 80 HCWs are present who have contact with patients in different wards. HCWs work in 8-hours shifts. HCWs confined to a single ward will work in the same ward during their next shift. Upon hospitalization patients can be admitted to both types of wards. In ICUs, 70% of the patients stay, on average, 1.5 days, with an ICU mortality of 2%. After ICU discharge, these patients stay, on average, seven days in regular wards, before hospital discharge. The remaining 30% of ICU-patients stay, on average, 10 days in ICU and have an ICU-mortality of 25%. ICU survivors remain hospitalized for, on average, 15 days in regular wards. These figures are based on real patient data from a multi-center ICU study in the Netherlands [11]. Length of stay is assumed to be independent of the colonization status. Apart from transfer from ICUs to wards, patients can be transferred between regular wards, from regular wards to ICUs, between ICUs, and between hospitals, all with different rates. Important parameters used are listed in Table 1.

**Table 1:** Parameters in the model

**Parameter**	**Value**	**Source**
Average length of stay in intensive care units	4 days	[17]
Average length of stay in regular ward	7 days	UMC
Admission from another hospitals	5%	UMC
Staff : patient ratio in intensive care units	1:1	UMC
Staff : patient ratio in regular ward	5:18	UMC
Staff : patient ratio of HCWs not restricted to single wards	~1:8.7	UMC
Duration of colonization in extramural population (mean)	370 days	[13,14]
Transmission risk intensive care units : regular ward	3:1	Assumption
Specificity of rapid diagnostic test	96%	[12]
Sensitivity of rapid diagnostic test	93%	[12]
Turnaround time of rapid diagnostic test	1 day	[12]
Specificity of conventional microbiological test (back-up test)	100%	Gold standard test assumed to be perfect
Sensitivity of conventional microbiological test (back-up test)	100%	Gold standard test assumed to be perfect
Turnaround time of conventional microbiological test (back-up test)	4 days	[12]

UMC-parameters estimated from data from University Medical Center Utrecht.

Patients are either carriers of MRSA or uncolonized and susceptible for colonization. Infection control interventions, however, are not based on the true colonization status, but on the available documentation of the colonization status only.

On hospital admission, MRSA carriage can be documented with a rapid diagnostic test that, for simplicity, provides a result in 24hours with sensitivity and specificity of 93% and 96% respectively [12]. Simultaneously, conventional microbiological tests, with assumed sensitivity and specificity of 100% and turn-around time of four days, are performed as back-up to adjust false test results of rapid tests. All patients should be screened on admission (i.e., universal screening), and we assume that compliance to this screening scenario is 88% (based on UMCU data) [10]. MRSA carriage may also be detected by clinical cultures, which are processed with conventional microbiological methods.

Patients can acquire MRSA by two modes: The first mode occurs via the hands of HCWs, which may have become contaminated after contact with a colonized patient. Appropriate hand hygiene will clean hand contamination and, therefore, hand contamination is typically short-lived. As a consequence, the probability of transmission via hands of temporary colonized HCWs is proportional to the fraction of colonized patients in the unit. The second acquisition mode is through persistently colonized HCWs, e.g., with carriage in the nares. This type of colonization is not short-lived and is not eradicated through hand hygiene. We assume that the risk for HCWs to become persistently colonized is proportional to the number of patients being colonized.

HCWs and patients may lose MRSA carriage in the extramural community after a median time of 256 days (mean of 370 days) [13,14]. Importantly, there is no patient-to-patient transfer of MRSA in the community, which limits our analyses to so-called hospital-acquired MRSA.

Most analyses are performed in settings with high endemicity levels of MRSA, i.e., in absence of any intervention specifically targeted at MRSA, the equilibrium patient prevalence of MRSA-carriage is around 14% and 40% in hospitals and ICUs, respectively. A medium endemicity level of around 6% and 20% in hospitals and ICUs, respectively, was analysed as well. A medium endemicity level is most realistic [15]. However, an MRSA-prevalence of 20% is not uncommon [16]. Transmission parameters in regular wards and ICUs were chosen to obtain these patient prevalences of MRSA and to obtain a prevalence of persistently colonized HCWs of 1%, 5% or 10%, while 10%, 30% or 50% of the acquisitions in patients can be ascribed to persistently colonized HCWs (see Additional file 1). As the feedback loop, (i.e. colonized patients who are discharged and readmitted) is included in our model we obtain the MRSA admission prevalence as a result of the chosen transmission parameters.

Note that we do not specify 1) hand hygiene compliance levels, 2) cohorting levels, 3) environmental cleaning protocols, 4) the use of single/multi bed rooms, 5) the frequency of contact between patients and HCW, and other factors influencing MRSA transmission. The effectiveness of interventions depends on the prevalence and relative importance of transmission modes only, and not directly on the aforementioned parameters. For instance, a high hand hygiene compliance with a low cohorting level will have the same effect on transmission as a low compliance with a high cohorting level. On top of the dynamics of MRSA as described in this section, we model intervention strategies, as described below, to address our research questions.

### Interventions

We consider two control strategies applied to patients with documented MRSA-carriage, and one applied to HCWs:

a) Isolation reduces both the likelihood for colonized patients to transmit MRSA and the likelihood for uncolonized patients (when isolated) to acquire colonization. The efficacy of isolation ranges from 0% (no effect of isolation) to 100% and is modelled as a multiplication factor (0 to 1) to transmission rates. Isolation with suboptimal efficacy could be considered to resemble strategies in which patients are not isolated in single-bed rooms, but in which other barrier precautions, e.g., gloves and gowns, are used instead. The number of beds available for patient isolation is unlimited, which allows quantification of the isolation needs for each intervention. Isolation measures are initiated when MRSA carriage (or infection) is documented. Isolation will be discontinued when screening cultures do not yield MRSA.

b) Decolonization of patients occurs a fixed number of days after the start of decolonization therapy. Until that time, or if decolonization is unsuccessful, the infectivity of a treated individual remains unaffected. If patients are discharged before the treatment is completed, the treatment will be continued extramurally. The efficacy of decolonization is denoted as the percentage of patients in which decolonization is successful. Decolonization is initiated on the same day that MRSA-carriage is documented. A successfully decolonized patient is immediately susceptible for acquisition of MRSA. If not specified otherwise, decolonization occurs instantaneously.

c) Decolonization of HCWs is assumed to be 100% efficacious and occurs, for simplicity, instantaneously. We explore the effects of decolonizing all staff with frequencies ranging from monthly to annually. After decolonization, HCWs are immediately susceptible for acquisition of MRSA.

Simulations for which we report 95% credibility intervals are always based on 1000 1,000 independent runs of the stochastic simulation model. Mean values can be based on 50 independent runs. We define the effectiveness of an intervention a time after the intervention has been implemented as the mean relative reduction in the hospital-wide MRSA prevalence.

## Results

### Comparing patient isolation and decolonization

**Figure 1:**
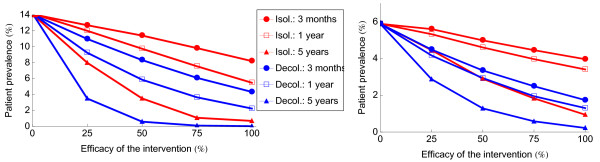
**Patient prevalence level of MRSA as function of the efficacy of patient decolonization or isolation after 3 months, 1 year and 5 years.** 88% of the patients are screened upon hospital admission. Carriers are either decolonized (red lines) or isolated (blue lines). The left figure corresponds to a high endemicity level, the right figure to a medium endemicity level.

With similar levels of efficacy, decolonization is more effective than patient isolation (Figure 1). Decolonized patients cannot reintroduce MRSA when readmitted to the hospital, which interrupts the so-called feedback loop. Another benefit of decolonization is that the nosocomial patient prevalence of MRSA decreases faster as compared to isolation strategies, because decolonization decreases the number of colonized patients in the hospital directly while isolation only prevents new acquisitions while patients in isolation are still colonized Nosocomial MRSA patient prevalence will decrease slower with a lower efficacy of control measures. With a lower efficacy of decolonization and isolation, the difference in patient prevalence between both interventions decreases during the first months after implementation. Three months after the start of interventions, the absolute difference in nosocomial MRSA patient prevalence between decolonization and isolation is 3.9% with 100% efficacy and 3.7%, 3.1% and 1.7% with 75%, 50% and 25% efficacy, respectively with a high endemicity level (14% hospital-wide) and 2.2%, 2.0%, 1.6% and 1.1% with an efficacy of isolation of 100%, 75%, 50% and 25% with a medium endemicity level (6% hospital-wide). On longer time scales, though, differences in patient prevalence between interventions show opposite trends. After 5 years the difference is 0.7% with 100% efficacy, and 1%, 2.9% and 4.5% with 75%, 50% and 25% efficacy, respectively. With a medium patient prevalence endemicity level, the difference is 0.7% with 100% efficacy, and 1.2%, 1.6% and 1.6% with 75%, 50% and 25% efficacy, respectively (see Figure 1).

Using an arbitrary goal for the ultimate patient prevalence of 0.3%, this goal will be reached in 5 years if the efficacy of patient decolonization exceeds 75% (Figure 1). For isolation, the patient prevalence will decrease slower. Even with a 100% efficacy the patient prevalence will be 1.5% after five years and the patient prevalence will be 2.3% and 5.8% with 75% and 50% efficacy respectively.

The effect of non-instantaneous decolonization of patients and HCWs is discussed in the Additional file 1.

### Role of health care workers

**Figure 2:**
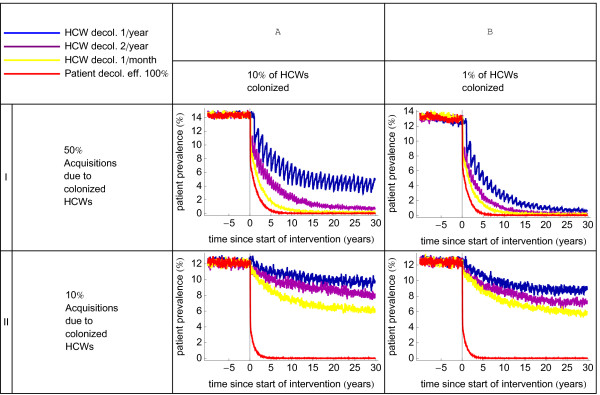
**The effects of health care worker decolonization on the patient prevalence level of MRSA.** Scenarios I and II, correspond to the relative importance of persistently colonized health care workers (HCW) in the spread of MRSA (being 50% and 10 %, respectively) in the endemic situation. Scenarios A and B correspond to different values for the percentage of persistently colonized HCWs. Results are based on 50 runs of the stochastic simulation model. The lines represent the average hospital-wide MRSA patient prevalence, starting from the baseline scenario of an average patient prevalence of 14% (high endemicity level). The red line represents the patient prevalence with patient decolonization (100% efficacious ) and the other lines represent the patient prevalence with health care worker decolonization (100% efficacious) performed once per year (blue), twice per year (purple) and every month (yellow).

The role of persistently colonized HCWs is composed of two aspects: the percentage of HCWs being persistently colonized (which depends on the probability for a non-colonized HCW to acquire colonization from a colonized patient) and the likelihood per colonized HCW to act as a source. Due to these different aspects few highly infectious persistently colonized HCWs may spread MRSA to the same number of patients as many persistently colonized HCWs who are individually less prone to spread MRSA. The benefits of decolonizing HCWs importantly depend on these parameters. Of note, this does not include their role as vectors with temporarily contaminated hands, which was considered as patient-to-patient transmission. We have evaluated the dynamics of the MRSA patient prevalence for several values of both aspects. The proportion of HCWs being persistently colonized ranged from 1% to 10%, and proportions of patient acquisitions resulting from persistently colonized HCW ranged from 10% to 50%. We quantified the effects of monthly, biannually and yearly decolonization of HCWs (Figure 2 and Additional file 1: Figure S1). The largest benefit from HCW decolonization is achieved when few persistently colonized HCWs are responsible for a large proportion of acquisitions. Naturally, monthly decolonization of HCWs is more effective than biannual and annual decolonization, but always less effective than decolonization of patients with documented MRSA carriage with an efficacy of 100%.

In practice, though, decolonization of patients will be less often successful than decolonization of HCWs [8]. We have, therefore, determined how efficacious patient decolonization should be to achieve the same MRSA patient prevalence (in 15 years after the start of the intervention) as 100% efficacious HCW decolonization (Additional file 1: Table S1 in the additional file). The maximum efficacy needed is 55%-68% for scenarios in which 50% of acquisitions result from persistently colonized HCWs. Yet, when persistently colonized HCWs are responsible for 10% of all acquisitions, efficacy of patient decolonization need only be 8%-9%. With a medium endemic prevalence, it will take more time before HCWs become persistently colonized. Therefore, interventions targeted at HCWs become more effective in settings with a lower patient prevalence (data not shown).

**Figure 3:**
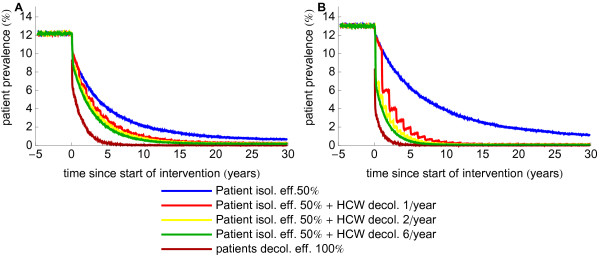
**Effect of combining patient isolation with decolonization of health care workers.** The two graphs correspond to scenarios with minimum effect of decolonization of HCWs (**A**) (10% of HCWs are persistently colonized and responsible for 10% of acquisitions ) and maximum (**B**) (1% of HCWs is persistently colonized and responsible for 50% of acquisitions). The effect of patient decolonization (100% efficacious) is added for comparison.

As HCW decolonization has been used in combination with patient isolation in several countries, we also investigated the effects of perfect periodical decolonization of HCWs in combination with patient isolation (with 50% efficacy). In Figure 3 we have depicted the most extreme scenarios (i.e., 10% prevalence among HCWs being responsible for 10% of acquisitions, and 1% of HCWs being responsible for 50% of acquisitions) in settings with higher endemicity level . As expected, the additional benefit of HCW decolonization is much higher in the latter scenario (Figure 3) but differences between decolonization frequencies are relatively small. The effect of the combined intervention in settings with medium endemicity level is shown in the additional file (Additional file 1: Figure S2).

**Figure 4:**
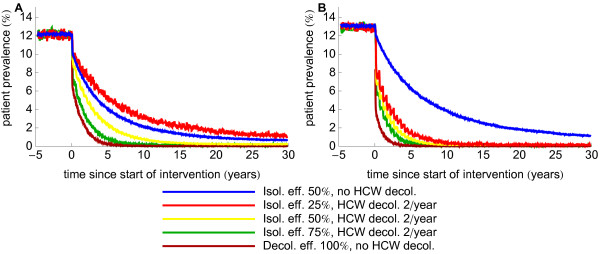
**Effect of the patient isolation efficacy when combined with biannual decolonization of health care workers.** The two graphs correspond to scenarios with minimum effect of decolonization of HCWs (**A**) (10% of the HCWs are persistently colonized and responsible for 10% of acquisitions) and maximum (**B**) (1% of the HCWs is persistently colonized and responsible for 50% of acquisitions). The efficacy of HCW decolonization is 100%. Lines with patient decolonization (100% efficacious) and only isolation (50% efficacious) are added for comparison.

The additional benefit of HCW decolonization is also influenced by the efficacy of patient isolation. Again using the two extremes as illustration, the benefit of adding HCW decolonization is much less sensitive to the efficacy of patient isolation when few HCWs are responsible for many acquisitions (Figure 4 for settings with a high endemicity level and Additional file 1: Figure S3 for a medium endemicity level).

In real life, multiple interventions are usually applied simultaneously. We, therefore, also determined the incremental effect of HCW decolonization to patient isolation and decolonization together. When isolation efficacy of patients with MRSA is 50% and decolonization efficacy among patients would be 90%, additional effects of HCW decolonization will be small, even for the `best-case scenario` of monthly decolonization with 1% of HCW being persistently colonized and responsible for 50% of acquisitions (data not shown). When the efficacy of patient decolonization is only 10% and isolation efficacy is 50%, monthly decolonization of HCWs will only substantially reduce MRSA patient prevalence in the extreme scenario with 1% of the HCW being persistently colonized and responsible for 50% of the acquisitions.

## Discussion

We have used a mathematical model to investigate the effects of isolation strategies for patients and of decolonization for patients and HCWs. Our findings demonstrate that – with similar levels of efficacy - patient decolonization is more effective than patient isolation and that active decolonization of persistently colonized HCWs only has a significant impact if a considerable proportion (e.g., 50% or more) of the MRSA acquisitions by patients can be ascribed to persistently colonized HCWs.

Our analyses clearly illustrate the two processes that determine the potential role of persistently colonized HCWs in MRSA transmission. One of these parameters, the proportion of HCWs being colonized, can easily be determined. Reported point-prevalence rates of HCW colonization in the nares range from <0.1% in Dutch hospitals with low endemic levels of MRSA [17] to 5-6% in hospitals with high endemic levels [18-21]. The other parameter, though, the relative contribution of these colonized HCWs for MRSA acquisition, is much more difficult to quantify, as both extensive screening among patients and HCWs and genotyping to demonstrate genetic similarities of MRSA isolates would be needed. Despite multiple, usually anecdotal, reports about MRSA carriage in HCWs, (as reviewed in [7]), this parameter has to the best of our knowledge never been quantified.

Naturally, the relative effects of HCW decolonization depend on the parameters used in the model. For instance, at lower endemic levels of MRSA the effects of HCW decolonization would be relatively higher. However, the dependency of two parameters, the fraction persistently colonized HCWs and the percentage of acquisitions resulting from them, remains important in all settings and estimation of these parameters in clinical settings will allow more precise determination of the effectiveness of HCW decolonization in reducing nosocomial MRSA-transmission.

Several studies have attempted to quantify the effects of bacterial eradication therapies in hospitalized patients [22,23]. In a systematic review, nasal application of mupirocin had, as compared to placebo, an estimated pooled relative risk of failure to eradicate nasal *S. aureus* carriage after one week of 0.10 (0.07-0.14), and effects were similar for patients and healthy subjects as well as in studies including only MSSA or both MSSA and MRSA carriers [8]. In a recent study, a combined approach of universal screening of MRSA carriage with PCR testing, followed by topical decolonization with mupirocin and isolation precautions for carriers, was associated with a 69.6% reduction in the aggregate hospital-associated MRSA disease incidence [24]. However, in the latter study, as in most studies in the systematic review, several interventions were tested simultaneously, hampering accurate quantification of the effects of decolonization.

In a Spanish intensive care burn unit topical application of vancomycin in the nose, oropharynx and intestines was evaluated in an observational before-after study during nine years [25]. Although no data are presented about the decolonization efficacy on a patient level, acquisition rates and average endemic patient prevalence levels were 80% lower with vancomycin use.

Another option, which we did not investigate, would be to restrict HCW decolonization to outbreak settings only. This strategy could lower the decolonization frequency of HCWs, especially when outbreaks are rare. However, the effectiveness of this strategy strongly depends on the definition of an outbreak and the sensitivity of detecting outbreaks.

Our analysis of non-instantaneous decolonization in the Additional file 1 is limited to patients. Indeed, instantaneous decolonization of HCWs may always be achieved in practice by temporary dismissal of known colonized HCWs and by replacing those by uncolonized ones.”

Although decolonization of patients seems, at least theoretically, an effective measure, these benefits should be balanced with potential adverse events. Topical use of mupirocin and antibiotics are considered safe, but selection of antibiotic resistance remains a potential threat. Especially the use of topical vancomycin should be carefully judged, as vancomycin is one the few remaining antibiotics available for intravenous treatment of MRSA infections.

Naturally, the model used is a simplification of reality. For instance, there are many specialized hospital wards with different patient populations and different patient transfer rates to other wards. Also, the susceptibility of patients to acquire MRSA will differ. Furthermore, we assumed that length of stay was not affected by colonization status, that all HCWs work in shifts of 8 hour, that direct transmission of MRSA between HCWs did not occur and that HCWs could not acquire persistent colonization outside hospital settings, e.g. from their colonized homes or families. Also, isolation was assumed to be equally efficacious in all isolated patients, which may not be true if the number of isolation beds available is limited. Finally, we did not explicitly model resistance development as a result of decolonization strategies. Our findings should, therefore, not be interpreted as a definitive argument in favour of widespread use of antibiotics for controlling the nosocomial spread of MRSA, but more as an illustration that different approaches might be more effective than our current strategies. Furthermore, we have identified research targets that could be pursued in epidemiological studies that are needed to further quantify the potential benefits of HCW decolonization.

## Conclusions

Based upon a theoretical framework, we have identified the scenarios in which decolonization of persistently colonized HCWs, either as a stand-alone measure or when added to interventions targeted at colonized patients, can significantly improve MRSA control in health care settings. In general, decolonizing HCWs becomes more beneficial when their carriage rates decrease and – simultaneously – their contribution to patient acquisition (per colonized HCW) increases.

Yet, decolonization of MRSA carriage among patients will be more efficacious than decolonization of HCWs in most scenarios with high endemicity levels, even for a low decolonization efficacy among patients. Furthermore, patient isolation, albeit conceptually less efficient than patient decolonization, will also be more effective than HCW decolonization. Note that if decolonization therapy in patients would not eradicate MRSA carriage, but only suppresses infectiousness by lowering the bacterial load, decolonization is conceptually similar to patient isolation as both reduce infectiousness without interrupting the feedback loop of colonized patients being readmitted. Considering the continuously rising patient prevalence levels of MRSA and the repeatedly reported failures of isolation policies to control its spread, our findings support further evaluation of pharmacological (and other) strategies to actively achieve eradication of MRSA carriage in patients.

## Abbreviations

HCW: Health care worker; ICU: Intensive care unit; MRSA: Methicillin-resistant *Staphylococcus aureus*.

## Competing interests

The authors have no conflict of interest.

## Authors’ contributions

TVG performed the simulation experiments, TVG and MCJB wrote the computer code. MCJB and MJMB designed the experiments. TVG drafted the manuscript. MCJB and MJMB helped to draft the manuscript. All authors read and approved the final manuscript.

## Pre-publication history

The pre-publication history for this paper can be accessed here:

http://www.biomedcentral.com/1471-2334/12/302/prepub

## Supplementary Material

Additional file 1 **Figure S1.** The effects of health care worker decolonization on the patient prevalence level of MRSA. **Figure S2.** The effects of combining patient isolation with 100% efficacious decolonization of health care workers. **Figure S3.** The effects of the patient isolation efficacy when combined with biannual 100% efficacious decolonization of health care workers. **Table S1.** Efficacy of patient decolonization needed to be equally effective as decolonization of health care workers. Click here for file
