# Supplementary material for: Decolonization of patients and health care workers to control nosocomial spread of methicillin-resistant Staphylococcus aureus: a simulation study
Source: BMC Infect Dis. 2012 Nov 14;12:302. doi: 10.1186/1471-2334-12-302 (PMC3526562; doi:10.1186/1471-2334-12-302)
Supplement: Additional file 1 — Figure S1. The effects of health care worker decolonization on the patient prevalence level of MRSA. Figure S2. The effects of combining patient isolation with 100% efficacious decolonization of health care workers. Figure S3. The effects of the patient isolation efficacy when combined with biannual 100% efficacious decolonization of health care workers. Table S1. Efficacy of patient decolonization needed to be equally effective as decolonization of health care workers. [file 1471-2334-12-302-S1.doc]

# Supplementary material to

# Decolonization of patients and health care workers to control nosocomial spread of methicillin-resistant *Staphylococcus aureus*: a simulation study

by

T.V. Gurieva, M.C.J. Bootsma, M.J.M. Bonten

**Additional Figures**


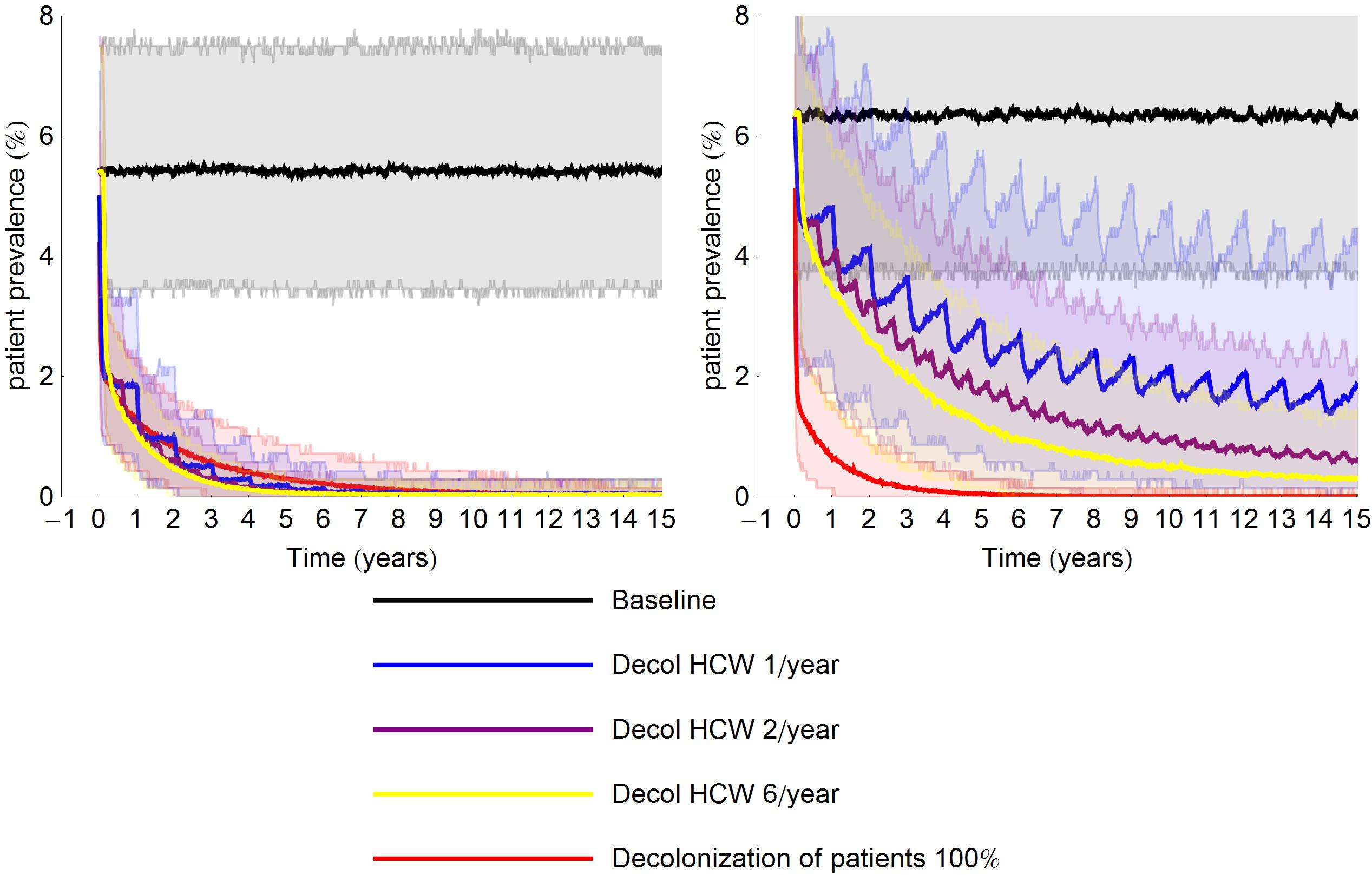


**Figure S1. The effects of health care worker decolonization on the patient prevalence level of MRSA.** The left figure corresponds to a relative importance of persistently colonized health care workers (HCW) on the spread of MRSA before interventions of 50%, the right figure to 10%. 5% percent of the HCWs are persistently colonized. Results are based on 1000 runs of the stochastic simulation model. The lines represent the average hospital-wide patient prevalence level of MRSA, starting from the baseline scenario of an average patient prevalence of 6%. The red line represents patient decolonization (100% efficacious). The other lines represent health care worker decolonization (100% efficacious) performed once per year (blue), twice per year (purple) and every month (yellow). Shaded areas correspond to 95% credibility intervals.


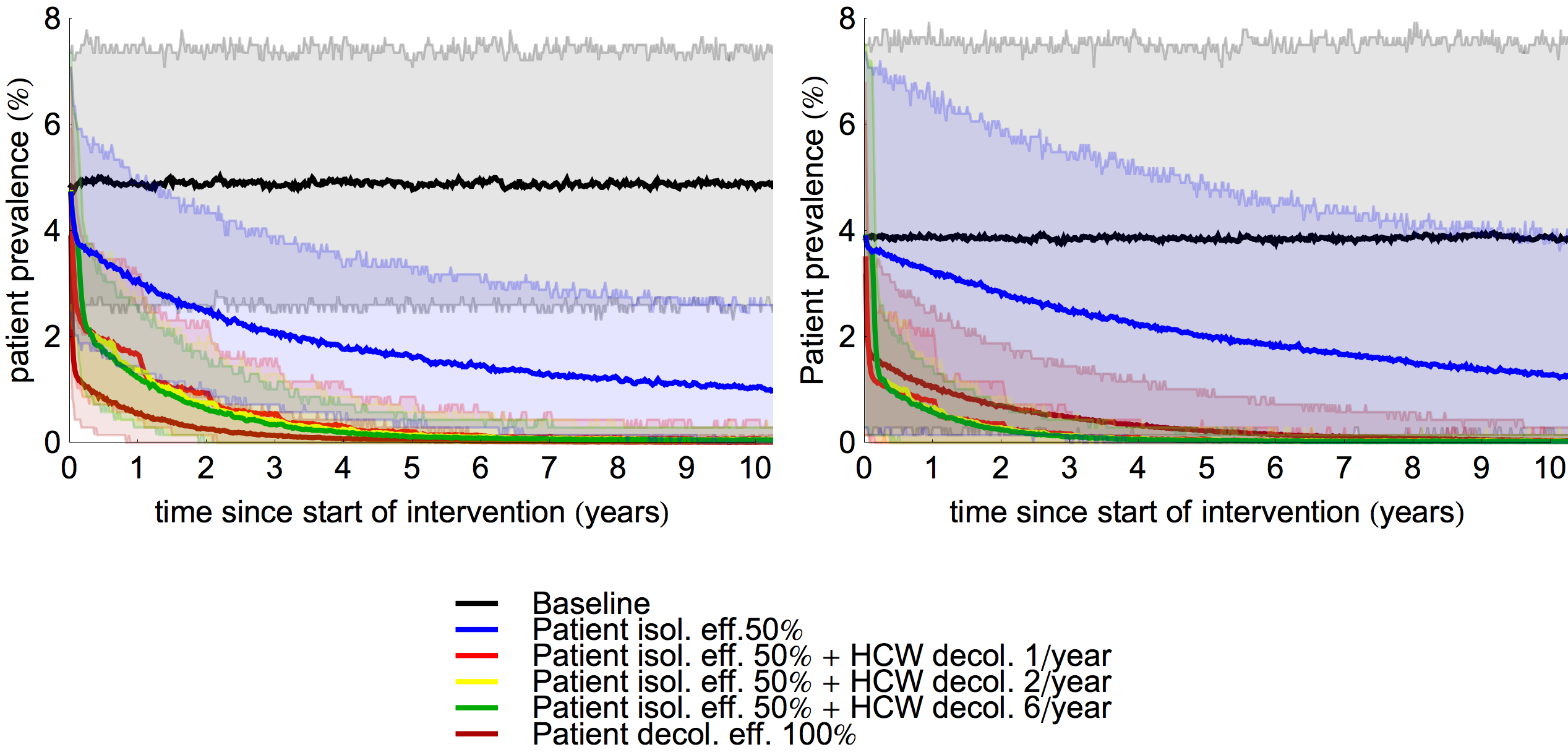


**Figure S2. The effects of combining patient isolation with 100% efficacious decolonization of health care workers.** The lines represent the average hospital-wide patient prevalence level of MRSA, starting from a medium endemic setting. The effect of decolonization of HCWs is minimal in the left figure (10% of the HCWs are persistently colonized and they are responsible for 10% of the acquisitions in the endemic situation) and maximal in the right figure (1% of the HCWs are persistently colonized and they are responsible for 50% of the acquisitions in the endemic situation). The effect of patient decolonization (100% efficacious) is added to compare these strategies. Shaded areas correspond to 95% credibility intervals.


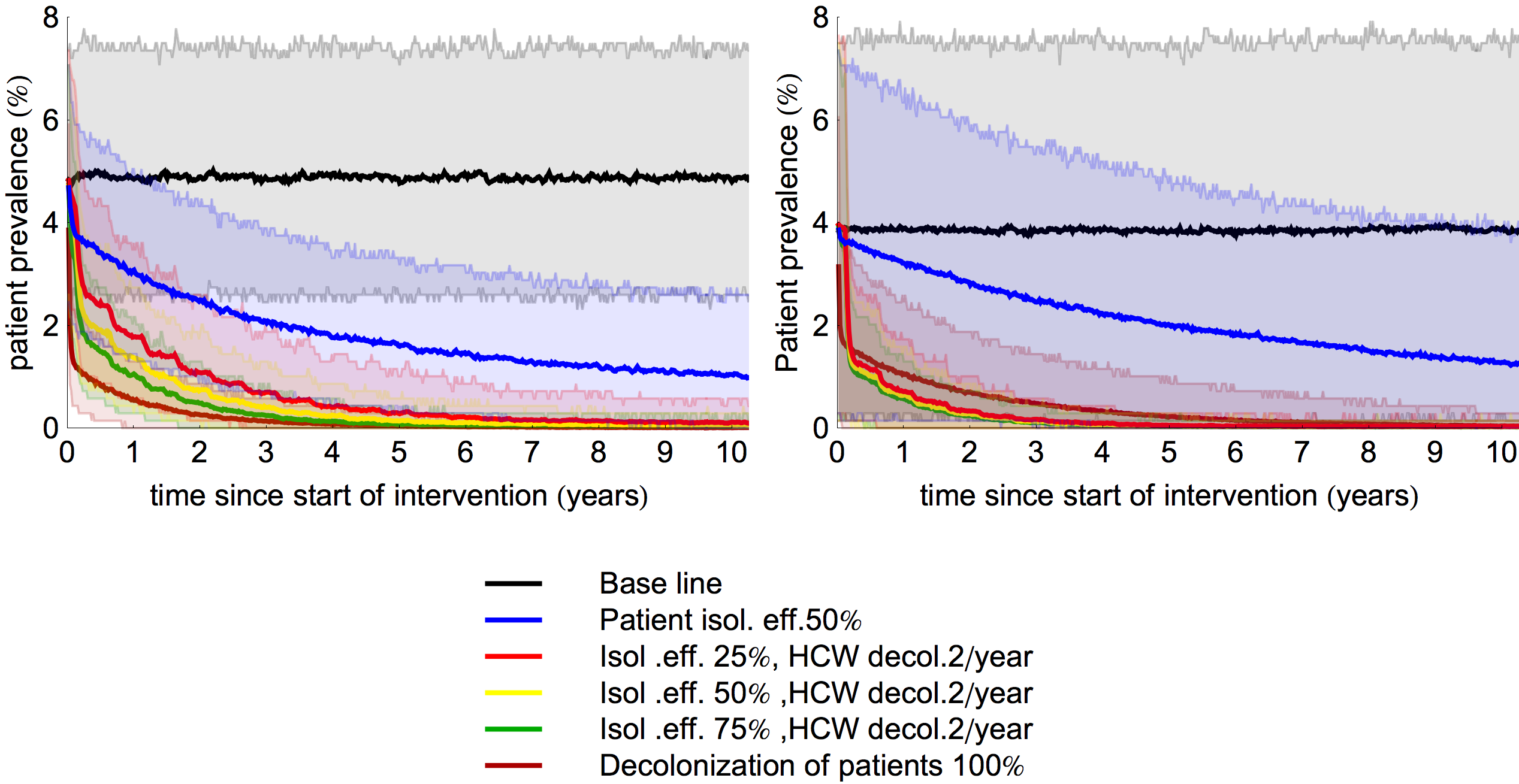


## Figure S3. The effects of the patient isolation efficacy when combined with biannual 100% efficacious decolonization of health care workers. The lines represent the average hospital-wide patient prevalence level of MRSA, starting from the baseline scenario of a medium endemic settings. The effect of decolonization of HCWs is minimal in the left Figure (10% of the HCWs are persistently colonized and they are responsible for 10% of the acquisitions in the endemic situation),and maximal in the right Figure (1% of the HCWs are persistently colonized and they are responsible for 50% of the acquisitions in the endemic situation). Lines with patient decolonization (100% efficacious) and only isolation (50% efficacious) are added to compare the strategies. Shaded areas correspond to 95% credibility intervals.

## Non-instantaneous decolonization

Where isolation can be achieved quickly, the decolonization process may take several days. When decolonization works not instantaneously, isolation will initially be more effective. For instance, isolation will reduce MRSA prevalence more effectively in the first 2 months if decolonization takes 10 days or more. However, even if decolonization takes that long, decolonization will ultimately be more effective. Here we assumed a constant time till decolonization, which is the worst-case scenario compared to all other distributions for the time till decolonization with the same mean (data not shown).We did not explicitly analyze non-instantaneous decolonization of HCWs. Yet, instantaneous decolonization of HCW can be realized, for example, by temporary dismissal of known colonized HCWs and by replacing those by uncolonized ones.

**Parameterization.**

We choose the patient prevalence of MRSA hospital-wide and in ICUs as well as the fraction HCWs who are persistently colonized and which fraction of the acquisitions of MRSA by patients are due to persistently colonized HCW. We varied 4 transmission parameters in our simulation code to obtain these desired values. These 4 parameters are 1) the susceptibility of HCWs for acquisition of persistent colonization, 2) the infectivity of persistently colonized HCWs, 3) the susceptibility for MRSA acquisitions of patients (increased susceptibility increases the acquisition rate of all modes) and 4) the relative difference in transmission rates in ICUs and other wards. We used a gradient based algorithm to obtain the best parameters, where we ran our coded for 50 years after a burn-in period for a given set of parameters. We started the algorithm at several initial values to avoid local minima. However, because our model is stochastic in nature, the obtained parameters may lead to slightly different values as desired for the patient prevalence of MRSA hospital-wide and in ICUs and the fraction HCWs who are persistently colonized and which fraction of the acquisitions of MRSA by patients are due to persistently colonized HCW .

## Table S1 - Efficacy of patient decolonization needed to be equally effective as decolonization of health care workers. We assume that health care worker (HCW) decolonization is 100% effective and is performed once a year, twice a year or every month. We have determined how efficacious universal screening followed by decolonization of known carriers should be in order to achieve the same hospital-wide MRSA patient prevalence (in 15 years after the start of the intervention) as HCW decolonization. Before the start of the intervention, 1%, 5% or 10% of the HCW are persistently colonized, while they are responsible for 10%, 30% and 50% of all MRSA acquisitions by patients.

|  | HCW decolonization | 10% of HCW colonized | 5% of HCW colonized | 1% of HCW colonized |
| --- | --- | --- | --- | --- |
| 50%  Acquisitions due to colonized HCW | Once per year | 28% | 32% | 25% |
|  | Once per half year | 43% | 47% | 45% |
|  | Once per month | 65% | 68% | 55% |
| 30%  Acquisitions due to colonized HCW | Once per year | 15% | 16% | 15% |
|  | Once per half year | 23% | 22% | 20% |
|  | Once per month | 32% | 30% | 27% |
| 10%  Acquisitions due to colonized HCW | Once per year | 4% | 4% | 4,5% |
|  | Once per half year | 6% | 6% | 6% |
|  | Once per month | 9% | 8% | 8% |
